# Effects of *Hint1* deficiency on emotional‐like behaviors in mice under chronic immobilization stress

**DOI:** 10.1002/brb3.831

**Published:** 2017-09-23

**Authors:** Liankang Sun, Peng Liu, Fei Liu, Yuan Zhou, Zheng Chu, Yuqi Li, Guang Chu, Ying Zhang, Jiabei Wang, Yong‐hui Dang

**Affiliations:** ^1^ First Affiliated Hospital Xi'an Jiaotong University Xi'an China; ^2^ College of Medicine & Forensics Key Laboratory of the Health Ministry for Forensic Medicine Key Laboratory of Environment and Genes Related to Diseases of the Education Ministry Xi'an Jiaotong University Health Science Center Xi'an China; ^3^ Clinical Research Center of Shaanxi Province for Dental and Maxillofacial Diseases College of Stomatology Xi'an Jiaotong University Xi'an China; ^4^ Qi De College Xi'an Jiaotong University Xi'an China; ^5^ Zong Lian College Xi'an Jiaotong University Xi'an China; ^6^ Department of Pharmaceutical Sciences School of Pharmacy University of Maryland Baltimore MD USA

**Keywords:** brain‐derived neurotrophic factor, chronic immobilization stress, haplo‐insufficient effect, hippocampus, histidine triad nucleotide‐binding protein 1, sex specific

## Abstract

**Background:**

Histidine triad nucleotide‐binding protein 1 (HINT1) is regarded as a haplo‐insufficient tumor suppressor and is closely associated with diverse neuropsychiatric diseases. Moreover, HINT1 is related to gender‐specific acute behavior changes in schizophrenia and in response to nicotine. Stress has a range of molecular effects in emotional disorders, which can cause a reduction in brain‐derived neurotrophic factor (BDNF) expression in the hippocampus, resulting in hippocampal atrophy and neuronal cell loss.

**Methods:**

This study examined the role of HINT1 deficiency in anxiety‐related and depression‐like behaviors and BDNF expression in the hippocampus under chronic immobilization stress, and investigated whether the sex‐specific and haplo‐insufficient effects exist in emotional‐like behaviors under the same condition.

**Results:**

In a battery of behavior tests, the results of the control group, not exposed to stress, showed that knockout (KO) and heterozygosity (HT) of *Hint1* had anxiolytic‐like and antidepression‐like effects on the male and female mice. However, both male and female *Hint1*‐KO mice showed elevated anxiety‐related and antidepression‐like behavior under chronic immobilization stress; moreover, both male and female *Hint1*‐HT mice displayed elevated anxiety‐related behavior and increased depression‐like behavior under chronic immobilization stress. There were no significant differences in general locomotor activity between *Hint1*‐KO and ‐HT mice and their wild‐type (WT) littermates. *Hint1*‐KO mice under basal and chronic immobilization stress conditions expressed more BDNF in the hippocampus than did *Hint1*‐HT and WT mice; overall, there were no significant sex differences in emotional‐like behaviors of *Hint1*‐KO and ‐HT mice. Additionally, Hint1‐HT mice showed haplo‐insufficient effects on emotional‐like behaviors under basic conditions, rather than under chronic immobilization stress.

**Conclusions:**

Both male and female HINT 1 KO and HT mice had a trend of anxiolytic‐like behavior and antidepression‐like behavior at control group. However, both male and female HINT1 KO mice showed elevated anxiety‐related and antidepression‐like behavior under chronic immobilization stress; moreover, both male and female HINT1 HT mice displayed elevated anxiety‐related behavior and increased depression‐like behavior under chronic immobilization stress.

## 1 INTRODUCTION

Mood and anxiety disorders are among the most common mental health conditions, resulting in functional disability, and major depressive disorder (MDD), with a lifetime risk of 20%–25% in women and 7%–12% in men has already created an enormous financial burden for modern human societies. Approximately 60% of people with MDD have comorbid anxiety disorder (Belmaker & Agam, 2008; Krishnan & Nestler, 2008). Comorbid anxiety and depression generally involve more severe symptoms, a continuous course of illness, and greater functional disability than either anxiety or depression alone (Funke et al., 2016; Moscati, Flint, & Kendler, 2016). Although these psychiatric disorders involve complex interactions between genetic and environmental factors, the etiology and the underlying molecular mechanism are still largely unclear (Finn, Rutledge‐Gorman, & Crabbe, 2003; Gispen‐de Wied & Jansen, 2002). Pharmacological agents used as antidepressants, such as selective serotonin reuptake inhibitors, are effective treatments, but up to 20% of patients completely fail to respond to standard interventions, and nearly 60% cannot achieve an adequate response (Block & Nemeroff, 2014). Moreover, a large proportion of patients have a poor prognosis; thus, it is urgent to explore the nosogenesis of anxiety and depression further at the molecular and cellular level, in order to develop more effective treatments.

Early life stress events are closely related to depression, and stressful events seem to play an important role in the onset, progression, and treatment effects of MDD and anxiety disorder (Fone & Porkess, 2008; Kim et al., 2011). Stress activates neuroendocrine systems and affects the activity of the relative brain area, causing physiological and behavioral responses (Chrousos, 2009; Ulrich‐Lai & Herman, 2009). The most commonly activated neuroendocrine system is the hypothalamus–pituitary–adrenal (HPA) axis, which plays important roles in the response to stressful conditions (Herman et al., 2003; Lupien, McEwen, Gunnar, & Heim, 2009). Activation of the HPA axis by stress can influence the expression of brain‐derived neurotrophic factor (BDNF) in the hippocampus. Hippocampal atrophy is also closely related to diverse neuropsychiatric diseases, including MDD, anxiety disorders, bipolar disorder, and schizophrenia (Buehlmann et al., 2010; Faul, Erdfelder, Lang, & Buchner, 2007; Geuze, Vermetten, & Bremner, 2005; Kempton et al., 2011; Shepherd, Laurens, Matheson, Carr, & Green, 2012). BDNF plays an essential role in neurogenesis and neuroplasticity and is implicated in depression (Buchmann et al., 2013; Karege et al., 2002; Lang & Borgwardt, 2013; Martinowich, Manji, & Lu, 2007). Stress experiences can reduce the level of BDNF in the hippocampus, leading to atrophic morphological change.

Previous studies have verified that the histidine triad nucleotide‐binding protein 1 (HINT1) is also distributed throughout the limbic system (Baumann & Bogerts, 1999; Liu, Puche, & Wang, 2008), and HINT1 belongs to the histidine triad protein super family, which possesses a conserved binding sequence motif HisXHisXHis (X = hydrophobic amino acid) (Klein et al., 1998). HINT1 was first recognized as the protein kinase C inhibitor‐1 (PKCI‐1) (Dang, Liu, Chen, Guo, & Wang, 2014). In humans, the HINT1 protein encoding gene is located on chromosome 5q31.2, and is widely expressed in multiple tissues, such as the liver, kidneys, and central nervous system (Jackson, Wang, Barbier, Chen, & Damaj, 2012; Liu et al., 2008; Zimon et al., 2012). At first, HINT1 gained attention as a tumor suppressor. Su et al. (2003) have reported that deletion of *Hint1* in mice promotes cell growth and carcinogenesis. Not only *Hint1*‐knockout (KO) mice but also *Hint1*‐heterozygous (HT) mice are more susceptible to induction of mammary and ovarian carcinogenesis by 7,12‐dimethylbenz[a]anthracene (DMBA) than wild‐type (WT) mice, which suggests that HINT1 is a haplo‐insufficient tumor suppressor (Li, Zhang, Su, Santella, & Weinstein, 2006). HINT1 also participates in the pathogenesis of hepatic fibrosis and diabetes (Chu, Fu, Meng, Zhou, & Zhang, 2013; Wu et al., 2013).

Interestingly, in recent studies, accumulating evidence from human postmortem analysis, research on mouse behavior, and anatomical studies has demonstrated that HINT1 is associated with diverse neuropsychiatric diseases. In a microarray analysis, *HINT1* mRNA expression was lower in the dorsolateral prefrontal cortex of patients who suffer from schizophrenic and bipolar disorder than healthy controls (Elashoff et al., 2007; Vawter et al., 2001, 2004). *HINT1* is also associated with abuse of drugs, such as nicotine (Jackson et al., 2011). Studies on the distribution of HINT1 in the brain have shown that it is expressed in neurons of the cerebral cortex and limbic system, which are anatomically related to mood disorders (Baumann & Bogerts, 1999; Benes & Berretta, 2001; Liu et al., 2008; Zhang & Reynolds, 2002). Knockout of *Hint1* in mice has antidepressant and anxiolytic‐like effects and elevated corticosterone levels in plasma (Barbier & Wang, 2009). However, Varadarajulu and colleagues have reported that *Hint1‐*KO mice showed increased anxiety‐like behavior (Varadarajulu et al., 2011). Additionally, the correlations between schizophrenia and HINT1 are sex specific (Chen et al., 2008; Jackson et al., 2012; Vawter et al., 2004), and nicotine‐mediated acute behavior changes also show different effects in males and females (Jackson et al., 2012). Nonetheless, it remains unknown whether HINT1 involved in the regulation of anxious and depressed behaviors under chronic immobilization stress is sex specific or haplo insufficient.

We speculated that HINT1 may be related to BDNF expression in the limbic system. However, it is not yet known whether *HINT1* knockout can affect BDNF expression in the hippocampus under chronic stress conditions. Thus, we considered whether *Hint1* deficiency would have effects on the emotional‐like behaviors of mice, as well as on the expression of BDNF in the hippocampus under chronic immobilization stress (CIS), and whether such effects would be influenced by gender and gene dosage (haplo‐insufficient effects). We used male and female mice that were homozygous mutant (KO), heterozygous mutant (HT), or wild type (WT) for *Hint1* in a CIS model. We then used a battery of behavioral tests to examine changes in emotional‐like behavior, and also explored BDNF protein expression in the hippocampus of these mice.

## 2 MATERIALS AND METHODS

### 2.1 Animal

The *Hint1*‐KO mouse model was previously generated in the Columbia University Health Science animal facility by Haiyang li and Bernard Weinstein as described (Li et al., 2006). Mice with three genotypes (homozygous mutant, heterozygous mutant, and their WT littermates) were the offspring of heterozygous breeding. The genetic background of the *Hint1*‐KO mice, ‐HT mice, and their WT littermates was initially 96% 129SvJ (Su et al., 2003). Mice were housed 4‐6/cage and allowed free access to water and food under standard laboratory conditions, in a temperature‐controlled (temperature: 23°C) environment, with a 12‐hr light/dark cycle, with lights on 8:00 a.m. Male and female mice of 10–12 weeks, of the three genotypes, including *Hint1*‐KO mice, ‐HT mice, and their WT littermates were used in this study.

We observed that *Hint1*‐KO and ‐HT mice display normal physiological growth and development and inconspicuous behaviors under home‐cage conditions. There were no significant differences in body weight in *Hint1*‐KO, ‐HT, or WT mice, which were weighed weekly from week 4 to week 12 (data are not shown).

All experimental protocols were approved by the Animal Care and Use Committee of Xi^,^an Jiaotong University.

### 2.2 Genotype identification

To identify genotypes of all types of progeny, genomic DNA was extracted from the tails of the mice, at the time of weaning (about 4 weeks), and was analyzed by PCR as described (Li et al., 2006). The forward primer 5′‐GCC TGA AGA ACG AGA TCA GC‐3′ and the reverse primer 5′‐CGC CCC AGT TAG TTA GT CAG‐3′ generated a 285‐bp product from the mutant allele, while the forward primer 5′‐GCC CCC TGT AAA GTG GAG AC‐3′ and the reverse primer 5′‐CGC CCC AGT TAG TTA GT CAG‐3′ produced a 339‐bp product from the wild‐type allele. Heterozygous mice produced both the 285‐bp and 339‐bp product.

### 2.3 Chronic immobilization stress

In order to create a reproducible animal model of chronic stress‐induced anxiety‐like and depression‐like behaviors, mice of six groups (3 genotypes × 2 genders) were subjected to CIS, and were restrained in a plastic cylinder with a height of 12 cm and diameter of 3 cm for 1 hr in the afternoon once daily, for 20 days, while the six negative control groups (3 genotypes × 2 genders) were handled in the same manner as the stressed animals, but without exposure to CIS. The 12 groups consisted of six experimental groups (3 genotypes × 2 genders) and six negative control groups without any stress (3 genotypes × 2 genders). There were 7–9 mice for each group; these animals were exposed to a battery of behavioral tests, starting on the 15th day of immobilization stress.

### 2.4 Behavioral testing

In order to investigate the effects of *Hint1*knockout on emotional‐like behaviors in mice under CIS, adult male and female *Hint1*‐KO mice, ‐HT mice, and their WT littermates were exposed to a number of paradigms estimating locomotor activity, anxiety‐related behavior, and depression‐like behavior (Cryan & Holmes, 2005). The animals were brought to the testing room at least 30 min before each experiment. There were 12 groups, as described above, for a series of behavior tests. For each group, behavioral tests were performed in the following order: Open‐field test (OFT), Elevated plus maze (EPM), Tail suspension test (TST), and Forced swimming test (FST). All the behavioral tests were conducted between 9 a.m. and 12 a.m., and were sorted by stress intensity from weak to strong, with a 48‐hr interval among the adjacent tests, and with a 12‐hr interval after the last immobilization. The results of all behavior tests were recorded and the relevant data were automatically collected using a video‐tracking system (SMART, Panlab SL, Barcelona, Spain).

#### 2.4.1 Open‐field test

The OFT was used to measure locomotor activity and explorative behavior, by assessing the rodents’ drive to explore unfamiliar environments. Briefly, mice were placed individually into a 45 × 45 × 45 cm open‐field chamber and tested for 1 hr. The data were recorded by SMART. The open field was divided into a central field (center, 20 × 20 cm) and a peripheral field. The total distance travelled in 1 hr was measured. After each trial, the chamber was thoroughly cleaned with water containing detergents, followed by 80% ethanol, and dried with a tissue to avoid olfactory influences on subsequent tests.

#### 2.4.2 Elevated plus maze

The EPM utilized a plus‐shaped apparatus to detect anxiety‐related behavior and was performed as described elsewhere (Lister, 1987). Briefly, the EPM consisted of two closed arms (25 × 5 × 20 cm) and two open arms (25 × 5 cm) connected by a central platform (5 × 5 cm), and was elevated 50 cm from the ground. Mice were placed in the central part of the platform, which is at the cross of the open and closed arms, and were permitted to explore the maze for 6 min. The data recorded included the number of entries mice made into the open arms, the number of entries made into the closed arms, the total time spent in the open arms, and the total time spent in the closed arms, and were analyzed by SMART. The proportion of time spent in the open arms was calculated as the “time spent in the open arms” × 100/(“the time spent in the open arms” + “the time spent in the closed arms”). After each test, the EPM was thoroughly cleaned with the same method as OFT.

#### 2.4.3 Tail suspension test

Mice were suspended by their tails to the edge of a shelf 80 cm above the ground. The tail was secured to the shelf by adhesive tape placed approximately 1 cm from the tip of the tail. Immobility time was recorded using SMART during a 6‐min session.

#### 2.4.4 Forced swimming test

Depression‐like and stress‐coping behavior was measured by means of the FST (Cryan & Holmes, 2005; Touma et al., 2008). Briefly, mice were placed in a Plexiglas cylinder (25 cm height × 15 cm diameter) containing 15 cm of water (22 ± 1°C) for 6 min. The behavior of mice and the immobility time during the 6‐min session were recorded using a video‐tracking system (SMART).

### 2.5 Protein extraction and Western blot

Total proteins in mice hippocampi were extracted using RIPA Lysis Buffer (Beyotime, Guangzhou, China) and the concentration of proteins was determined using the BCA protein assay kit (Pierce, Rockford, USA) according to the manufacturer's instruction. Western blotting assays were performed as previously described (Zhang, Fang, & Wang, 2015). Primary antibodies were anti‐BDNF (dilution 1:1,000; #sc‐546, Santa Cruz, Dallas, TX, USA) and anti‐β‐actin (dilution 1:1,000; #sc‐8432, Santa Cruz). The protein expression was visualized using enhanced chemiluminescence (Millipore, Billerica, MA, USA). Images were captured using the ChemiDoc XRS imaging system (Bio‐Rad, Hercules, CA, USA) and Quantity One image software was used for the densitometric analysis of each band. β‐actin was used as an internal loading control.

### 2.6 Statistical analysis

All data were expressed as mean ± standard error of mean (*SEM*), and we used SPSS18.0 software for data processing and analysis. For data of OFT of distance moved every 10 min, repeated‐measures ANOVA with between‐subjects factors was used to analyze within‐subject differences (control vs. CIS) and between‐subject differences (genotype), statistical differences in other behavioral studies were performed using three‐way analysis of variance (ANOVA) with treatment, sex, and genotype as the between‐subject factors, and significant results were further analyzed using a post hoc multiple comparison (Bonferroni post hoc test). Statistical significance was set at *p* < .05.

## 3 RESULTS

### 3.1 Effects of Hint1 deficiency and CIS on locomotor activity

**Figure 1:**
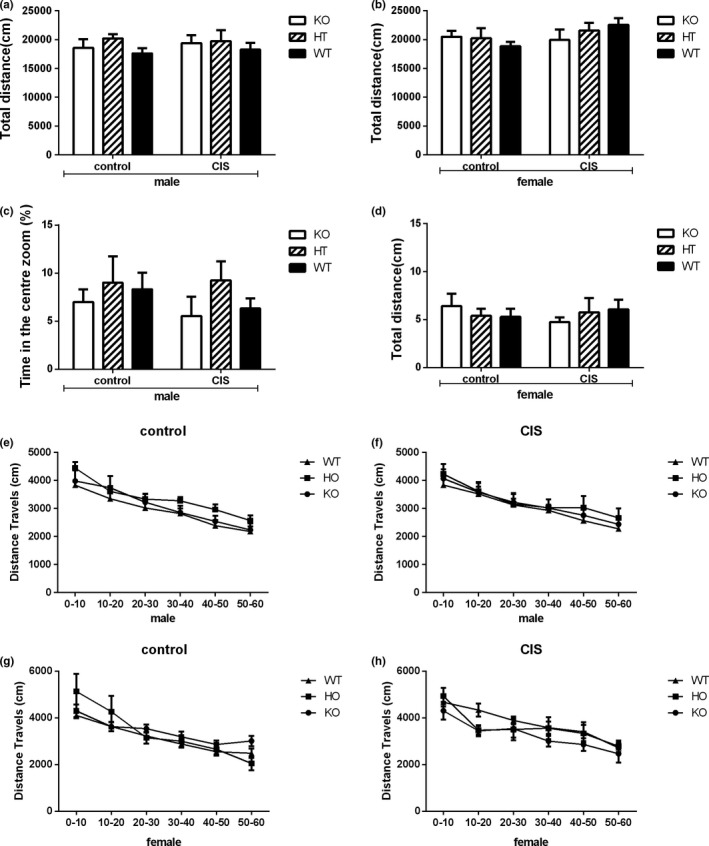
The locomotor activity of *Hint1*‐KO and ‐HT mice and their WT littermates under basal conditions, with no stress, and CIS conditions in the open‐field test (OFT). (a) The total distance moved by male mice in 60 min; (b) The total distance moved by female mice in 60 min; (c) The time spent in the center zoom (%) in the first 10 min in male mice; (d) The time spent in the center zoom (%) in the first 10 min in female mice; (e) Distance moved every 10 min in male mice under basal condition; (f) Distance moved every 10 min in male mice under chronic immobilization stress, (g) Distance moved every 10 min in female mice under basal condition; (h) Distance moved every 10 min in female mice under chronic immobilization stress. All data are expressed as the mean ± *SEM* (*n* = 7–9 for each group). Control: basal conditions, with no stress; CIS, chronic immobilization stress. KO,* Hint1*‐knockout mice; HT,* Hint1*‐heterozygous mice; WT, wild type

The OFT was used to evaluate the locomotor activity and exploratory behavior in Hint1‐KO and ‐HT mice as compared to their WT littermates under basal or chronic immobilization stress (CIS) conditions. The statistical analysis of total distance of OFT showed that there were no significant main effects of gender (*F*_1,72_ = 2.417, *p* = .124), no significant main effects of genotype (*F*_2,72_ = 0.331, *p* = .719), no significant main effects of intervention (*F*_1,72_ = 1.338, *p* = .251), no significant gender × genotype (*F*_2,72_ = 0.570, *p* = .568), no significant gender × intervention (*F*_1,72_ = 1.231, *p* = .271), no significant genotype × intervention (*F*_2,72_ = 0.730, *p* = .485), and no significant gender × genotype × intervention interactions (*F*_2,72_ = 0.406, *p* = .668). Similarly, there were no statistically significant differences with the time spend in the center zoom (%) in the first 10 min and distance moved every 10 min. Therefore, we found no significant differences in Hint1 deficiency and CIS on locomotor activity in OFT (Figure 1).

### 3.2 Effects of Hint1 deficiency and CIS on the anxiety‐related behavior

The EPM test was applied to detect anxiety‐related behavior in mice with complete or partial loss of Hint1 under basal or CIS conditions. The statistical analysis of the percentage of open‐arm time in the EPM showed that there were no significant main effects of gender (*F*_1,72_ = 1.039, *p* = .359), no significant gender × genotype (*F*_2,72_ = 1.062, *p* = .351), no significant gender × intervention (*F*_1,72_ = 0.098, *p* = .756), and no significant gender × genotype × intervention interactions (*F*_2,72_ = 0.042, *p* = .959). However, there were significant main effects of genotype (*F*_2,72_ = 8.945, *p* = .004), significant main effects of intervention (*F*_1,72_ = 55.239, *p* < .0001), and significant genotype × intervention interactions (*F*_2,72_ = 6.066, *p* = .004).

The statistical analysis of the number of entries into the open‐arm in the EPM showed that there were no significant main effects of gender (*F*_1,72_ = 1.073, *p* = .304), no significant main effects of genotype (*F*_2,72_ = 1.468, *p* = .237), no significant gender × genotype (*F*_2,72_ = 0.345, *p* = .709), and no significant gender × genotype × intervention interactions (*F*_2,72_ = 2.811, *p* = .067). However, there were significant main effects of intervention (*F*_1,72_ = 54.019, *p* < .0001), significant gender × intervention (*F*_1,72_ = 7.515, *p* = .008), and significant genotype × intervention interactions (*F*_2,72_ = 12.331, *p* < .0001). These statistical results of EPM revealed that there were no significant gender differences on anxiety‐related behavior in mice with Hint1 deficiency under basal or CIS conditions.

**Figure 2:**
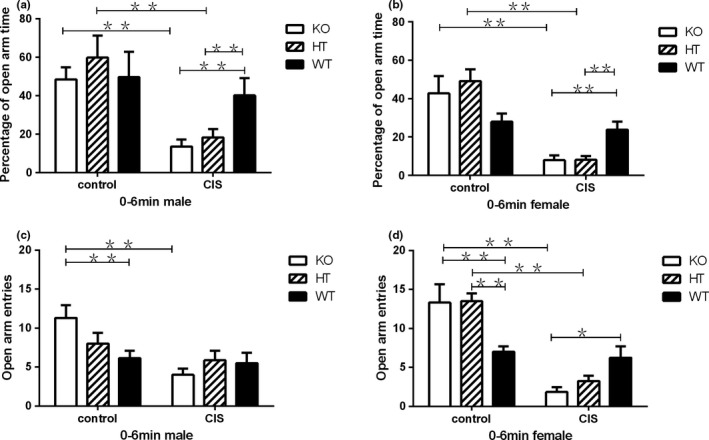
The anxiety‐related behaviors of *Hint1*‐KO and ‐HT mice and their WT littermates under basal conditions, with no stress, and CIS conditions in the elevated plus‐maze (EPM) test. (a) Percentage of open‐arm time of male mice, and (b) percentage of open‐arm time of female mice. The statistical analysis of the percentage of open‐arm time in the EPM showed that there were no significant main effects of gender (*F*_1,72_ = 1.039, *p* = .359), but significant main effects of genotype (*F*_2,72_ = 8.945, *p* = .004), significant main effects of intervention (*F*_1,72_ = 55.239, *p *< .0001), and significant genotype × intervention interactions (*F*_2,72_ = 6.066, *p* = .004). (c) Open‐arm entries of male mice, (d) open‐arm entries of female mice. The statistical analysis of the number of entries into the open arm in the EPM showed that there were no significant main effects of gender (*F*_1,72_ = 1.073, *p* = .304) and no significant main effects of genotype (*F*_2,72_ = 1.468, *p* = .237). However, there were significant main effects of intervention (*F*_1,72_ = 54.019, *p* < .0001), significant gender × intervention (*F*_1,72_ = 7.515, *p* = .008), and significant genotype × intervention interactions (*F*_2,72_ = 12.331, *p* < .0001). Values represent mean ± *SEM* (*n* = 7–9 for each group). *p < .05; **p < .01. Control: basal conditions, with no stress; CIS, chronic immobilization stress. KO,* Hint1*‐knockout mice; HT,* Hint1*‐heterozygous mice; WT, wild type

The post hoc multiple comparison analysis showed that compared with the control group at basal conditions, Hint1‐KO and ‐HT male and female mice significantly spent less time in the open‐arm after CIS (both *p* < .01, in Figure 2a,b). Both male and female Hint1‐KO and ‐HT mice showed a significant reduction in the percentage of open‐arm time as compared with their WT littermates under CIS conditions (*p* < .01).

At basal conditions, Hint1‐KO mice and Hint1‐HT female mice showed more entries into the open‐arm in the EPM test compared to WT mice (both *p* < .01; Figure 2c,d). As compared with the control group at basal conditions, Hint1‐KO mice and Hint1‐HT female mice had significantly lower numbers of entries in the open‐arm after CIS (both *p* < .01, in Figure 2c,d). Similarly, Hint1‐HT male mice had the same trend despite no significant statistical difference.

Taken together, these results showed that both male and female Hint1‐KO and ‐HT mice demonstrated elevated anxiety‐related behavior under CIS.

### 3.3 Effects of Hint1‐deficiency and CIS on the depression‐like behavior in TST

The immobility time of *Hint1*‐KO and ‐HT mice and their WT littermates in the TST was used to measure the depression‐like behavior. There were no significant main effects of gender (*F*_1,72_ = 0.675, *p* = .414) and no significant gender × genotype (*F*_2,72_ = 2.870, *p* = .063). However, there were significant main effects of genotype (*F*_2,72_ = 4.689, *p* = .012), significant main effects of intervention (*F*_1,72_ = 27.465, *p* < .0001), significant gender × intervention (*F*_1,72_ = 6.496, *p* = .013), and significant gender × genotype × intervention interactions (*F*_2,72_ = 3.574, *p* = .033).

**Figure 3:**
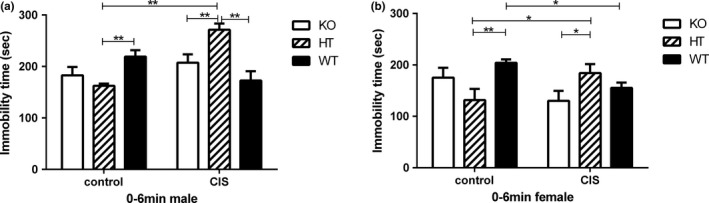
The depression‐like behaviors of *Hint1*‐KO and ‐HT mice and their WT littermates under basal condition, with no stress, and CIS conditions in the tail suspension test (TST). (a) Immobility time (s) of male mice, (b) immobility time (s) of female mice. There were no significant main effects of gender (*F*_1,72_ = 0.675, *p* = .414), but significant main effects of genotype (*F*_2,72_ = 4.689, *p* = .012), significant main effects of intervention (*F*_1,72_ = 27.465, *p* < .0001), significant gender × intervention (*F*_1,72_ = 6.496, *p* = .013), and significant gender × genotype × intervention interactions (*F*_2,72_ = 3.574, *p* = .033). In addition, the immobility time of *Hint1*‐HT male and female mice was significantly decreased under basal conditions, but increased under CIS. Both male and female *Hint1*‐KO mice tended to have decreased immobility time under basal conditions, while *Hint1*‐KO female mice also displayed a decreased immobility time under CIS. Values represent mean ± *SEM* (*n* = 7–9 for each group). *p < .05; **p < .01. Control: basal conditions, with no stress; CIS, chronic immobilization stress. KO,* Hint1*‐knockout mice; HT,* Hint1*‐heterozygous mice; WT, wild type

In the post hoc multiple comparison analysis, the immobility time of *Hint1*‐HT male and female mice was significantly decreased as compared with their WT littermates under basal conditions as represented in Figure 3a,b (*p* < .01), and both male and female *Hint1*‐KO mice tended to show decreased immobility time under basal conditions in the TST (Figure 3a,b). However, compared with Hint1‐HT mice in control group, both Hint1‐HT male and female mice also showed significantly increased immobility time after CIS (*p* < .01). Taken together, the TST results showed that both male and female Hint1‐HT mice had antidepression‐like behavior at basal conditions, and Hint1‐KO mice had the trend of antidepression‐like behavior at basal conditions. However, Hint1‐HT mice increased depression‐like behavior after CIS, while Hint1 knockout in female mice tended to have antidepression‐like effects.

### 3.4 Effects of Hint1 deficiency and CIS on the depression‐like behavior in FST

**Figure 4:**
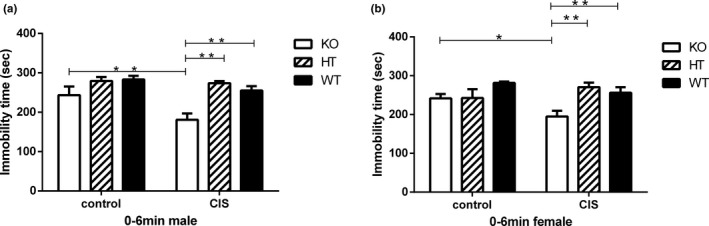
The depression‐like behaviors of *Hint1*‐KO and ‐HT mice and their WT littermates under basal conditions, with no stress, and CIS conditions in the forced swimming test (FST). (a) Immobility time (s) of male mice, (b) immobility time (s) of female mice. There were no significant main effects of gender (*F*_1,72_ = 0.365, *p* = .547), but significant main effects of genotype (*F*_2,72_ = 19.636, *p* < .0001) and significant main effects of intervention (*F*_1,72_ = 8.839, *p* = .004) and significant genotype × intervention interactions (*F*_2,72_ = 5.835, *p* = .004). In addition, both male and female *Hint1*‐KO mice exhibited a slightly decreased immobility time under basal conditions, and the immobility time was significantly decreased under CIS. *Hint1*‐HT female mice had a decreased immobility time under basal conditions, but displayed an increased immobility time under CIS. Values represent mean ± *SEM* (*n* = 7–9 for each group). *p < .05; **p < .01. Control: basal conditions, with no stress; CIS, chronic immobilization stress. KO,* Hint1*‐knockout mice; HT,* Hint1*‐heterozygous mice; WT, wild type

The results of effects of Hint1 deficiency and CIS on the depression‐like behavior in FST are shown in Figure 4. There were no significant main effects of gender (*F*_1,72_ = 0.365, *p* = .547), no significant gender × genotype (*F*_2,72_ = 1.008, *p* = .370), no significant gender × intervention (*F*_1,72_ = 1.185, *p* = .280), and no significant gender × genotype × intervention interactions (*F*_2,72_ = 0.330, *p* = .720). These results indicated that there was no significant gender difference on the depression‐like behavior in FST of Hint1‐deficiency mice under CIS. However, there were significant main effects of genotype (*F*_2,72_ = 19.636, *p* < .0001) and significant main effects of intervention (*F*_1,72_ = 8.839, *p* = .004) and significant genotype × intervention interactions (*F*_2,72_ = 5.835, *p* = .004). Therefore, both a genotype and intervention and their interactions effects occurred in the FST.

In the post hoc multiple comparison analysis, compared to the baseline of the control group, Hint1‐KO male and female mice had a significantly shorter immobility time after CIS (*p* < .01; Figure 4a,b). The immobility time of Hint1‐KO male and female mice was significantly shorter than that of Hint1‐HT mice or their WT littermates under CIS (*p* < .01; Figure 4a,b). Both male and female Hint1‐KO mice and Hint1‐HT female mice exhibited a slight trend for decreased immobility time under basal conditions in the FST (Figure 4a,b). Although there were no statistical differences, both male and female Hint1‐HT mice showed a trend for increased immobility time as compared with Hint1‐KO mice or their WT littermates after CIS, which was consistent with the TST of Hint1‐HT mice.

Taken together, the results of FST showed that both male and female Hint1‐KO mice showed reduced depression‐like behavior as compared with their WT littermates under CIS. Both male and female Hint1‐HT mice showed an increased trend for depression‐like behavior under CIS.

### 3.5 BDNF expression in hippocampus of Hint1‐KO and ‐HT mice and their littermates

After finishing of the last behavior tests (FST), mice were sacrificed and total proteins were extracted from mouse hippocampi for Western blotting analysis of BDNF expression. The statistical analysis of BDNF expression showed that there were significant main effects of gender (*F*_1,72_ = 5.394, *p* = .022), significant main effects of genotype (*F*_2,72_ = 10.615, *p* < .0001), significant main effects of intervention (*F*_1,72_ = 7.965, *p* = .006), and no significant gender × genotype × intervention interactions (*F*_2,72_ = 0.062, *p* = .940).

**Figure 5:**
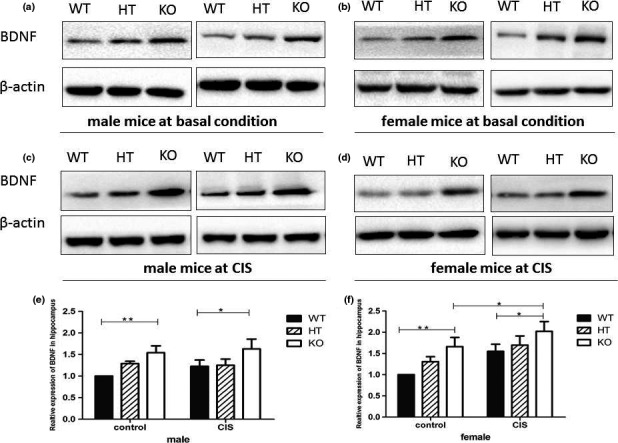
Brain‐derived neurotrophic factor (BDNF) expression in the hippocampus of *Hint1*‐KO and ‐HT mice and their WT littermates under basal conditions or under CIS, as determined by Western blotting. Representative images of BDNF expression in hippocampus under basal conditions in male mice (a) and female mice (b); representative image of BDNF expression in the hippocampus under CIS in male mice (c); and female mice (d); densitometric analyses of the relative expression of BDNF in the hippocampus of male mice (e); and female mice (f) by QuantityOne image analysis software. The BDNF results were normalized to β‐actin, and the value in the WT group under basal conditions (control group) was set as 100%. The statistical analysis of BDNF expression showed that there were significant main effects of gender (*F*_1,72_ = 5.394, *p* = .022), significant main effects of genotype (*F*_2,72_ = 10.615, *p* < .0001), significant main effects of intervention (*F*_1,72_ = 7.965, *p* = .006), and no significant gender × genotype × intervention interactions (*F*_2,72_ = 0.062, *p* = .940). In addition, *Hint1*‐KO mice not only in basic condition but also under chronic immobilization stress exhibited higher expression of BDNF in hippocampus than *Hint1*‐HT mice and their WT littermates; *Hint1*‐HT mice had higher levels of expression of BDNF than their WT littermates, but lower than that of *Hint1*‐KO mice, not only under basic condition but also under CIS. Values represent mean ± *SEM* (*n* = 7–9 for each group). *p < .05; **p < .01. Control: basal conditions, with no stress; CIS, chronic immobilization stress. KO,* Hint1*‐knockout mice; HT,* Hint1*‐heterozygous mice; WT, wild type

Our results (Figure 5) showed that *Hint1*‐KO mice, under both basic and CIS conditions, exhibited higher expression of BDNF in the hippocampus than *Hint1*‐HT mice and their WT littermates, which correlated with the reduced depression‐like behavior of *Hint1*‐KO. *Hint1*‐HT mice also showed a higher level of BDNF expression than their WT littermates, but a lower level than that of *Hint1*‐KO mice under basic as well as under CIS conditions.

## 4 DISCUSSION

This study investigated whether *Hint1* gene deficiency has effects on emotional‐like behaviors in mice and the expression of BDNF in their hippocampus under CIS conditions, to determine whether the effects of *Hint1* deficiency has sex‐specific and haplo‐insufficient effects on emotional‐like behaviors of mice under CIS. On the basis of integrating all the data results, we got the following conclusions from the whole point of view. In a battery of behavior tests, under basal conditions, and as compared to the WT control group, both male and female *Hint1*‐KO and ‐HT mice generally showed anxiolytic‐ and antidepressant‐like effects. However, both male and female *Hint1*‐KO mice showed increased anxiety‐related behavior but antidepressant‐like behaviors under CIS; moreover, both male and female *Hint1*‐HT mice displayed elevated anxiety‐related behavior and increased depression‐like behavior under CIS. Some results did not reach statistical differences but had a trend of statistical differences, small samples may be one of the possible reason. Furthermore, in the OFT, there were no significant differences in the general locomotor activity of *Hint1*‐KO, ‐HT, and WT littermates, which were in line with the previous findings of Varadarajulua and colleagues, and Barbier and colleagues (Barbier et al., 2007; Varadarajulu et al., 2011). Interestingly, the mood behavior results found by Varadarajulua et al. were in contrast to the findings of Wang. Varadarajulua reported that *Hint1*‐KO mice demonstrated increased anxiety‐related behavior and elevated emotional‐like arousal, as compared with WT mice, while the findings of Wang revealed that deletion of *Hint1* in mice demonstrated anxiolytic‐like and antidepressant‐like effects (Barbier & Wang, 2009). Previous explanations for these contradictory findings included different test settings, such as light intensities, the size of the compartments, the age and time of testing, etc. (Varadarajulu et al., 2011). Our findings of *Hint1*‐KO mice under basal conditions, with no stress, were in accordance with the findings of Wang. However, under CIS conditions, our results supported those of Varadarajulu. Accumulating evidence has shown that different stressful events play an essential role in the onset and progression of many psychiatric disorders, such as anxiety disorders and MDD (Charney & Manji, 2004; Lupien et al., 2009). Thus, these findings indicate that the degree of stress intensity may exert pivotal roles in mood behaviors of *Hint1*‐KO mice.

Early studies have demonstrated that HINT1 is a haplo‐insufficient tumor suppressor (Li et al., 2006). Whether haplo‐insufficient effects are also present in emotional‐like behavior remains unclear. Under basal conditions, both full and partial deletion of *Hint1* in mice tended to have anxiolytic‐like and antidepressant‐like effects on behavior. After CIS, *Hint1*‐HT mice showed elevated anxiety‐related behavior and increased depression‐like behavior, while *Hint1*‐KO mice displayed elevated anxiety‐related behavior and reduced depression‐like behavior. Therefore, our results showed that haplo‐insufficient effects may exist in the emotional‐like behaviors of *Hint1*‐HT mice under basal conditions, but not under CIS conditions. Moreover, we propose that the antidepressant‐like effects of knockout of *Hint1* in mice represent the genotype rather than the stress effect. The HPA axis commonly dysfunctions under stress conditions, and is involved in mood disorders (Dedovic, Duchesne, Andrews, Engert, & Pruessner, 2009; Jo, Zhang, Emrich, & Dietrich, 2015; Schule, Baghai, Eser, & Rupprecht, 2009). The finding of a trend toward anxiolytic‐like effects in the elevated anxiety‐like behavior in *Hint1*‐KO mice after CIS indicates that CIS plays an important role in changes in anxiety‐related behaviors, and that the underlying mechanism may involve activation of the HPA axis by stress. However, after CIS, the emotional‐like behaviors of *Hint1*‐HT mice changed markedly from a trend toward anxiolytic‐like effects and antidepressant‐like effects, to increased anxiety‐related and depression‐like behavior, implying that the underlying mechanism may be more complicated. Firstly, these results indicate that *Hint1*‐HT mice are more sensitive to a CIS stimulus for totally reverting emotional‐like behaviors from a tendency toward anxiolytic‐like effects and antidepressive‐like effects, to the elevated anxiety‐related and increased depression‐like behavior. However, *Hint1‐*HT mice had a higher expression of BDNF than their WT littermates, while it was lower than that of *Hint1*‐KO mice, not only under the basic condition but also under CIS. This indicated that the expression of BDNF in the hippocampus of *Hint1*‐HT mice may represent the effect of the gene (*Hint1*) and that other underlying molecular alterations under CIS may participate in these emotional‐like behavior changes. Secondly, *Hint1*‐HT mice in vivo still retain a certain amount of expression of HINT1 protein, thus, there may have been a physiological compensatory effect in the process of mouse growth and development. Furthermore, it remains unclear why reduced expression of HINT1 protein in *Hint1*‐HT mice in interaction with a stress stimulus caused changes in emotional‐like behavior, and this requires further study.

There are gender differences between men and women in terms of the occurrence, symptoms, and therapeutic responses of stress‐related mental disorders, such as MDD, which is twice as common in females than in males (Marcus et al., 2005). However, there are no gender differences in the rates of MDD at the puberty (Dalla, Pitychoutis, Kokras, & Papadopoulou‐Daifoti, 2010), which suggests that age may exert an important effect in the mental disorders with a gender difference. However, in our studies, we did not find significant sex differences overall in the emotional‐like responses to HINT1 KO and HT mice. The age of mice may exert important effects on emotional‐like behaviors that do not show a sex difference. Moreover, the neurobiology of mental disorders, such as anxiety and depression itself, is still not fully understood. Furthermore, there is still lack of relative basic research of developing animal models and drug treatments that are sensitive to sex differences. It is necessary to further investigate the mechanism of HINT1 associations with gender differences in mental disorders.

Stress can activate a large array of molecular effects in the brain, especially in the hippocampus, which then generate functional, structural, molecular, and behavior alterations (McEwen, 2001; Sapolsky, 2003). Current studies have implicated that depression is not only viewed as abnormalities in the monoaminergic systems but is also involved in the impairment of neural plasticity, neurotrophic factors, and dendritic complexity in the hippocampus (Duman & Monteggia, 2006), and BDNF gene transcriptional activity can influence the neuronal growth, plasticity, and neurogenesis, which is conducive to the therapeutic response to antidepressants. Our Western blot results showed that *Hint1*‐KO mice not only under basic conditions but also under chronic immobilization stress exhibited higher expression of BDNF in hippocampus compared with *Hint1*‐HT mice and their WT littermates, which agreed with the finding that *Hint1*‐KO mice displayed antidepression‐like behavior, not only under basal conditions but also under CIS.

Early studies have showed that PKC activity in brain lysates of *Hint1*‐KO mice was higher than in their WT littermates, and PKCγ protein isoform expression increased in *Hint1*‐KO mice (Varadarajulu et al., 2011). Moreover, elevated abundances of PKCγ were also detected in Hint1 haplo‐insufficiency tissue such as hippocampus, the cortex, etc. (Zhang et al., 2015). Some studies have revealed that intracellular signal transduction plays an important role in the pathophysiology of depression. One key set of mechanisms implicate kinases, such as protein kinases A (PKA) and C (PKC) (Pandey & Dwivedi, 2005). Protein kinases are critical elements of stimulus‐response coupling, which can phosphorylate the transcriptional factor cyclic AMP response element‐binding protein (CREB) (Hyman & Nestler, 1996). Thus, we speculated that elevated PKC activity in *Hint1*‐KO and ‐HT mice promoted the phosphorylation of CREB, which bound to the cAMP‐responsive element (CRE) sites at the promoter of *Bdnf*, enhancing the protein expression level of BDNF in the hippocampus. The high expression level of *Bdnf* in *Hint1* KO mice exerts antidepression effects not only under basal conditions but also under CIS. CIS resulted in changes in emotional‐like behavior in *Hint1*‐KO (from the trend for anxiolytic‐like behavior to the elevated anxiety‐related behavior) and HT mice (from the trend of anxiolytic‐like and antidepression‐like behaviors, to the elevated anxiety‐related behavior and increased depression‐like behavior), may through other underlying molecular mechanisms. Barbier reported that absence of HINT1 may involve dysregulation of postsynaptic dopamine transmission (Barbier et al., 2007). It is possible that such variation in dopamine transmission may exert important role in HINT1‐related emotional‐like behaviors alterations after CIS; therefore, the molecular mechanism still needs further clarification.

## CONFLICT OF INTEREST

None declared.
